# DNA index as prognostic factor in childhood acute lymphoblastic leukemia in the COG-TARGET database

**DOI:** 10.1186/s12885-021-08545-6

**Published:** 2021-07-15

**Authors:** Kun-yin Qiu, Xiong-yu Liao, Zhan-wen He, Ruo-hao Wu, Yang Li, Lu-hong Xu, Dun-hua Zhou, Jian-pei Fang

**Affiliations:** 1 Department of Paediatrics, Sun Yat-sen Memorial Hospital, Sun Yat-sen University, Guangzhou, 510120 People’s Republic of China; 2 Guangdong Provincial Key Laboratory of Malignant Tumor Epigenetics and Gene Regulation, Sun Yat-Sen Memorial Hospital, Sun Yat-Sen University, Guangzhou, 510120 People’s Republic of China

**Keywords:** ALL, Children, DNA index, chemotherapy, value

## Abstract

**Background:**

This study was aimed to evaluate the value of DNA index(DI) among pediatric acute lymphoblastic leukemia (ALL) treated on Children’s Oncology Group (COG) protocols between 2000 and 2015.

**Methods:**

Retrospective study were analysis among pediatric ALL patients from the TARGET dataset.

**Result:**

Totally, 1668 eligible pediatric patients were enrolled in this study. Of them, 993 are male and 675 are female with a median age of 7.6 years old. The median follow-up for those patients was 7.7 years (range 0.1–15.7 years). The probability of 15-year EFS and OS were reported to be 67.5 ± 3.1% and 78.3 ± 2.5%, respectively. BCR/ABL1 fusion gene affected the early treatment response and the survival of childhood ALL. Moreover, those patients with ETV6/RUNX1 fusion gene were also significantly associated with better EFS (*HR* = 0.6, 95% *CI* 0.4–0.8, *P* = 0.003) and OS (*HR* = 0.3, 95%*CI* 0.2–0.5, *P* < 0.001) compared to patients with no ETV6/RUNX1. On the contrary, BM NR on Day+ 29 showed a significant decrease in EFS (*HR* = 3.1, 95%*CI* 2.1–4.5, *P <* 0.001) and OS (*HR* = 1.7, 95%*CI* 1.1–2.8, *P =* 0.026).

Multivariate analysis showed that DI was significantly associated with better EFS and OS. The threshold effect of DI on poor outcome was significant after adjusting for potential confounders. The adjusted regression coefficient (Log RR) was 0.7 (95%*CI* 0.1–3.2, *P* = 0.597) for DI < 1.1 while 8.8 (95%*CI* 1.4–56.0, *P* = 0.021) for DI ≥ 1.2 and 0.0 (95%*CI* 0.0–0.8, *P* = 0.041) for 1.1 ≤ DI < 1.2. Generalized additive models revealed that the lowest rates of the adverse outcomes estimated to occur among DI between 1.1 and 1.2.

**Conclusion:**

For those childhood ALL treated on COG protocols between 2000 and 2015, ETV6/RUNX1 and BM NR were closely related to the prognosis. Moreover, the DI between 1.1 and 1.2 can serve as a significant cut-point discriminating the risk group, which indicated a favourable prognostic factor.

## Background

Acute lymphoblastic leukemia (ALL) is the most common hematological malignant tumor in children, accounting for 80% of childhood leukemia [1]. ALL is a malignant clonal disease of hematopoietic stem cells, which is a heterogeneous hematological disease caused by abnormal clone and proliferation of primitive and immature lymphocytes in bone marrow and peripheral blood. With the improvement of the understanding of molecular genetics and pathogenesis of ALL, combined with risk classification, multidrug intensive therapy and hematopoietic stem cell transplantation, the cure rate and survival outcome of children with leukemia have been significantly improved in recent years, but the pathogenesis and specific etiology are still unclear [2, 3]. So far, it has been found that cytogenetic changes play an important role in the occurrence and development, treatment and prognosis of childhood leukemia [4].Among these cytogentic findings, the DNA index (DI), which represents the DNA content of leukemic cells, has been considered an important prognostic factor for risk determination [5]. However, studies [6–9] on the best DI threshold affecting prognosis were published in 1980s, 1990s and 2008, while the latest study [10] in 2017 suggested the value of DI and its conventional cut-point should be re-evaluated in patients treated with the recent chemotherapy protocols. In this study, our purpose was to evaluate the value of DI on early treatment response and prognosis among 1668 pediatric ALL patients treated on Children’s Oncology Group (COG) chemotherapy protocols between 2000 and 2015, so as to provide evidence for assessment of risk stratification and differences in the intensities of chemotherapy in treatment protocol.

## Patients and methods

### Study participants

The clinical and laboratory data about pediatric ALL aged≤18 years old was downloaded from the Therapeutically Applicable Research to Generate Effective Treatments dataset (December 21, 2020). Between May 2000 and August 2015, 1990 patients were registered in the TARGET database: 50 were not eligible (second malignancy, *n* = 17; Down syndrome, *n* = 33), 272 were not assessable (inadequate information on diagnosis or treatment), and 1668 childhood ALL were enrolled in our study finally.The results published here are in whole based upon data generated by the TARGET (https://ocg.cancer.gov/programs/target) initiative, phs000218. The data used for this analysis are available at https://portal.gdc.cancer.gov/projects. The trial was approved by COG, and informed written consent was obtained from all study participants. All methods were performed in accordance with the relevant guidelines and regulations such as French-American-British (FAB) criteria in the manuscript. Chemotherapy protocols for childhood ALL included 9906, AALL0232, AALL0331 and AALL0434, the application of different treatment protocols were related to the update time of the protocols and the choice of researchers in different centers.

### Diagnostic studies

Bone marrow(BM) samples were transported overnight and processed at the blood analysis laboratory within 24 h. Diagnosis of ALL was based on French-American-British (FAB) criteria: less than 3% blasts positive for myeloperoxidase or Sudan black, and negative for nonspecific esterase [11]. Immunophenotyping was performed by flow cytometry (Epics XL; Beckman Coulter, Miami, FL) [12, 13]. For DI measurement, 300,000 to 500,000 mononuclear cells were stained with propidium iodide using automatic DNA staining equipment and analyzed by flow cytometry after two hours. DI was defined as the ratio of the number of mode channels of G0/G1 peak in tumor cells to that in normal cells [14]. Cytogenetic in situ analysis was performed by standard G-banding / fluorescence in situ hybridization (FISH) techniques. Hyperdiploid refers to a cell or individual that has one or more additional chromosomes or chromosome fragments in addition to the normal genome.Pro B-ALL expresses HLA-DR, TdT, and CD19, CD10, cytoplasmic immunoglobulin negative; Common ALL is characterized by the presence of CD10,cytoplasmic immunoglobulin negative; Pre B-ALL is characterised by the expression of cytoplamic immunoglobulin and CD 10; Mature B-ALL, was whose blast cells express surface antigens of mature B cells, including surface membrane immunoglobulin(SmIg+). They are typically TdT and CD34 negative and have L3 Morphology [15].

Complete remission (CR) was defined as non-leukemic signs, no leukemic cells detected in blood smears, active hematopoiesis in bone marrow, less than 5% of leukemic primordial cells, and normal cerebrospinal fluid (CSF). Bone marrow aspiration was examined on Day+ 29 at the end of phase IA. According to the dataset, the year of diagnosis is from 2000 to 2015. The last follow-up year was from 2000 to 2017.

### Statistical analysis

Baseline characteristics were presented as mean ± SD for continuous variables and as frequency (%) for categorical variables. Comparisons between groups were made using the chi-square test for categorical variables and analysis of variance or the Kruskal-Wallis test for continuous variables. To assess outcome, the following parameters were used: CR rate, event-free survival (EFS, defined as time between diagnosis and first event, including induction failure, relapse, progression or death of any cause), overall survival (OS, defined as time between diagnosis and death from any cause). EFS and OS were estimated by the Kaplan–Meier method and compared using the log-rank test. The univariate and multivariate logistic regression analyses were performed to identify risk factors of early treatment response and prognostic factors. A generalized additive model (GAM) with a spline smoothing function was applied to examine the relationship between DI and adverse clinical outcomes (adverse clinical outcomes, defined as time between diagnosis and first event, including induction failure, relapse, progression or death of any cause). Adjusted OR with 95% CIs were estimated to evaluate the association of DI and adverse clinical outcomes, which was with adjustments for gender, age, race, chemotherapy protocol, white blood cell(WBC); central nervous system(CNS) status, fusion gene, prednisone response, BM blasts on Day+ 29, immunophenotype and karyotype. All statistical analyses were performed using the IBM SPSS Statistics version 22.0, and EmpowerStats (http://www.empowerstats.cn/). A 2-tailed *p* < 0.05 was considered to be statistically significant in all analyses.

## Results

### Baseline characteristics of patients

Totally, 1668 eligible pediatric patients were enrolled in this study. The baseline characteristics of the eligible patients were presented in Table 1. Of them, 993 (59.5%) are male and 675 (40.5%) are female with a median age of 7.6 years old. According to the immunophenotype, 721 (43.2%) patients were diagnosed as B-precursor ALL. In addtion, Fusion gene was detected in 1307 patients via conventional G-banding analysis and the most common positive fusion gene was ETV6/RUNX1. Among the pediatric patients, the median of initial WBC was 33.7 × 10^9^/L (range 0.4–1306.0 × 10^9^/L), and the median DI was 1.0 (range 0.0–1.9).

**Table 1:** Baseline Characteristics of Study Participants

Characteristics	Number
Age(y), median(range)	7.6 (1–18)
Age group, years	
≥ 1, < 10	951 (57.0%)
≥ 10	717 (43.0%)
Gender, n(%)	
Male	993 (59.5%)
Female	675 (40.5%)
Race, n(%)	
White	1246 (74.7%)
Non-white	422 (25.3%)
WBC(×10^9^/L), median(range)	33.7 (0.4–1306.0)
WBC group, ×10^9^/L	
< 50	956 (57.3%)
≥ 50	712 (42.7%)
Immunophenotype, n(%)	
B-Precursor	721 (43.2%)
B Cell ALL	497 (29.8%)
T Cell ALL	242 (14.5%)
B precursor (Non-T, Non-B ALL)	208 (12.5%)
DI, median(range)	1.0 (0.51–1.9)
Karyotype, n(%)	
Normal	739 (44.3%)
hypodiploidy	98 (5.9%)
hyperdiploid	395 (23.7%)
NA	436 (26.1%)
Fusion gene, n(%)	
ETV6/RUNX1	213 (12.8%)
MLL	56 (3.4%)
TCF3/PBX1	92 (5.5%)
BCR/ABL1	41 (2.5%)
Negative	905 (54.3%)
Unknown	361 (21.6%)
CNS status, n(%)	
CNS1	1353 (81.1%)
CNS2	245 (14.7%)
CNS3	70 (4.2%)
Prednisone response, n(%)	
Good	1154 (69.2%)
Poor	514 (30.8%)
BM blasts on Day+ 29, n(%)	
CR	1625 (97.4%)
NR	43 (2.6%)
BM relapse, n(%)	
No	1359 (81.5%)
Yes	309 (18.5%)
CNS relapse, n(%)	
No	1555 (93.2%)
Yes	113 (6.8%)
Testes site of relapse, n(%)	
No	1657 (99.3%)
Yes	11 (0.7%)
Chemotherapy protocol, n(%)	
9906	222 (13.3%)
AALL0232	789 (47.3%)
AALL0331	415 (24.9%)
AALL0434	242 (14.5%)

WBC white blood cell, DI DNA index, CNS central nervous system, BM bone marrow, NR not remission

As a result, 222 patients accept 9906 protocol, 789 patients accept AALL0232, 415 patients received AALL0331 protocol and the remaining 242 patients received AALL0434 protocol. As for the early treatment response, 1154 patients responded well to prednisone on Day+ 8, while the remaining 514 patients did not respond well to prednisone. Most of the pediatric patients (*n* = 1625) achieved CR at the end of remission induction chemotherapy (Day + 29).

**Fig. 1:**
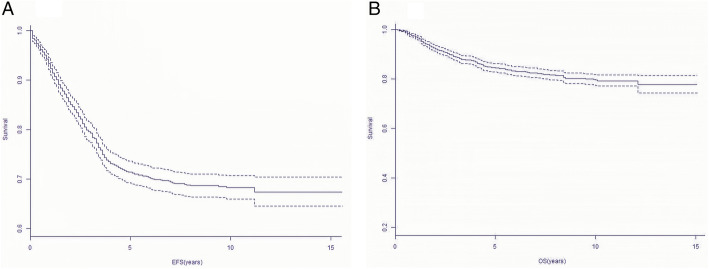
Ten-year Survival curves of all pediatric ALL patients. Probability of 15-year EFS for pediatric ALL patients. Probability of 15-year OS for pediatric ALL patients

The median follow-up for those patients was 7.7 years (range 0.1–15.7 years). Finally, 309 patients subsequently developed hematological relapse (presence of leukemic blasts > 25% in BM), 113 patients developed CNS relapse and testes site of relapse occurred in 11 children. The probability of 10-year EFS and OS were reported to be 67.5 ± 3.1% and 78.3 ± 2.5%, respectively (Fig. 1).

### Early treatment response

We conducted a univariate and multivariate analysis of the early treatment response on Day + 29 after induction chemotherapy among childhood ALL. Factors associated with a significantly elevated of early treatment response from univariate analysis were: age,WBC, immunophenotype, karyotype, fusion gene, prednisone response and chemotherapy protocol (Table 2). No association early treatment response was found with the gender, race, DI and CNS status. Results from the multiple regression analysis found that age ≥ 10 years(*OR* = 9.7, 95%*CI* 1.6–59.1, *P* = 0.014), B Cell ALL(*OR* = 0.2, 95%*CI* 0–0.7, *P* = 0.019), B precursor (Non-T, Non-B)(*OR* = 0.1, 95%*CI* 0–0.8, *P* = 0.034), hyperdiploid(*OR* = 0.2, 95%*CI* 0–0.6, *P* = 0.009), BCR/ABL1 fusion gene(*OR* = 7.6, 95%*CI* 2.4–24.3, *P <* 0.001), poor prednisone response (*OR* = 16.9, 95%*CI* 6.3–45.9, *P <* 0.001), AALL0232 protocol (*OR* = 0, 95%*CI* 0–0.5, *P =* 0.011) and AALL0434 (*OR* = 0, 95%*CI* 0–0.1, *P <* 0.001) protocol were the independent risk factors (Table 2).

**Table 2:** The correlations between various factors and early treatment response

Characteristics	Univariate analysis		Multivariate analysis	
	OR(95%*CI*)	*P* value	OR(95%*CI*)	*P* value
Age(y)	1.1 (1.0, 1.2)	0.004	0.9 (0.8, 1.0)	0.127
Age group				
≥ 1, < 10	Ref	< 0.001	Ref	0.014
≥ 10	3.2 (1.6, 6.1)		9.7 (1.6, 59.1)	
Gender				
Male	Ref	0.615	Ref	0.330
Female	1.2 (0.6, 2.2)		1.4 (0.7, 3.0)	
Race				
White	Ref		Ref	
Non-white	1.6 (0.8, 3.0)	0.146	1.6 (0.8, 3.3)	0.229
WBC	1.0 (1.0, 1.0)	< 0.001	1.0 (1.0, 1.0)	0.466
WBC group				
< 50	Ref	< 0.001	Ref	0.069
≥ 50	3.2 (1.7, 6.2)		2.5 (0.9, 6.8)	
Immunophenotype				
B-Precursor	Ref		Ref	
B Cell ALL	0.2 (0.1, 0.6)	0.003	0.2 (0.0, 0.7)	0.019
T Cell ALL	0.2 (0.0, 0.9)	0.032	1.0	
B precursor (Non-T, Non-B)	1.1 (0.5, 2.4)	0.773	0.1 (0.0, 0.8)	0.034
DI	0.2 (0.0, 3.1)	0.249	2.0 (0.0, 336.1)	0.790
Karyotype				
Normal	Ref		Ref	
hypodiploidy	0.9 (0.3, 2.9)	0.816	0.5 (0.1, 2.5)	0.384
hyperdiploid	0.2 (0.1, 0.7)	0.011	0.2 (0.0, 0.6)	0.009
NA	0.7 (0.3, 1.5)	0.347	0.7 (0.3, 1.6)	0.393
Fusion gene				
Negative	Ref		Ref	
ETV6/RUNX1	0.0 (0.0, Inf)	0.983	0.0 (0.0, Inf)	0.988
MLL	2.3 (0.7, 7.8)	0.194	1.4 (0.3, 5.8)	0.683
TCF3/PBX1	0.4 (0.1, 3.3)	0.426	0.9 (0.1, 7.1)	0.882
BCR/ABL1	12.9 (5.7, 29.7)	< 0.001	7.6 (2.4, 24.3)	< 0.001
Unknown	0.8 (0.3, 1.9)	0.598	1.0 (0.3, 2.8)	0.935
CNS status				
CNS1	Ref		Ref	
CNS2	1.4 (0.6, 3.1)	0.409	0.8 (0.3, 2.1)	0.624
CNS3	1.8 (0.6, 6.2)	0.319	1.0 (0.2, 4.7)	0.960
Prednisone response				
Good	Ref	< 0.001	Ref	< 0.001
Poor	18.3 (7.2, 46.9)		16.9 (6.3, 45.9)	
Chemotherapy protocol				
9906	Ref		Ref	
AALL0232	0.7 (0.3, 1.4)	0.291	0.0 (0.0, 0.5)	0.011
AALL0331	0.1 (0.0, 0.5)	0.003	0.2 (0.0, 3.0)	0.241
AALL0434	0.2 (0.0, 0.7)	0.018	0.0 (0.0, 0.1)	< 0.001

WBC white blood cell, DI DNA index, CNS central nervous system, BM bone marrow, NR not remission

### Multivariate analysis of prognostic factors

When we included DI with other risk factors in the Cox model, including age, gender, race, WBC, fusion gene, prednisone response, BM blasts Day+ 29, immunophenotype and karyotype as co-variables, we identified DI as an independent factor for both EFS and OS in pediatric patients with ALL (Table 3). DI was significantly associated with EFS (*HR* = 0.9, 95%*CI* 0.3–2.0, *P* = 0.048) and OS (*HR* = 0.1, 95%*C*I 0–0.5, *P* = 0.001). Those patients with ETV6/RUNX1 fusion gene were also significantly associated with better EFS (*HR* = 0.6, 95% *CI* 0.4–0.8, *P* = 0.003) and OS (*HR* = 0.3, 95%*CI* 0.2–0.5, *P* < 0.001) compared to patients with no.

**Table 3:** Multivariate analysis for EFS and OS among pediatric patients with ALL

Outcome	Variable	HR (95% *CI*)	*P* value
EFS	Age ≥ 10	0.9 (0.6, 1.5)	0.771
	Female	0.9 (0.7, 1.0)	0.087
	Non-white	1.0 (0.8, 1.2)	0.848
	WBC ≥ 50	1.3 (0.9, 1.7)	0.110
	B Cell ALL	0.2 (0.1, 0.2)	< 0.001
	DI	0.9 (0.3, 2.0)	0.048
	Hyperdiploid	0.8 (0.6, 1.0)	0.110
	ETV6/RUNX1	0.6 (0.4, 0.8)	0.003
	BCR/ABL1	1.6 (1.0, 2.5)	0.048
	PPR	1.3 (1.1, 1.6)	0.004
	BM NR on Day+ 29	3.1 (2.1, 4.5)	< 0.001
	AALL0434 protocol	0.3 (0.1, 1.2)	0.07
OS	Age ≥ 10	1.3 (0.7, 2.3)	0.457
	Female	0.9 (0.7, 1.1)	0.208
	Non-white	1.0 (0.8, 1.4)	0.727
	WBC ≥ 50	1.5 (1.0, 2.1)	0.026
	B Cell ALL	0.2 (0.1, 1.3)	0.054
	DI	0.1 (0.0, 0.5)	0.001
	Hyperdiploid	0.6 (0.5, 0.9)	0.015
	ETV6/RUNX1	0.3 (0.2, 0.5)	< 0.001
	BCR/ABL1	1.3 (0.7, 2.3)	0.367
	PPR	1.3 (1.0, 1.6)	0.055
	BM NR on Day+ 29	1.7 (1.1, 2.8)	0.026
	AALL0434 protocol	0.4 (0.2, 1.6)	0.125

WBC white blood cell, DI DNA index, CNS central nervous system, BM bone marrow, NR not remission

ETV6/RUNX1. On the contrary, BM NR on Day+ 29 showed a significant decrease in EFS (*HR* = 3.1, 95%*CI* 2.1–4.5, *P <* 0.001) and OS (*HR* = 1.7, 95%*CI* 1.1–2.8, *P =* 0.026).

### The value of DI cut-point among prognostic impact of pediatric ALL

**Fig. 2:**
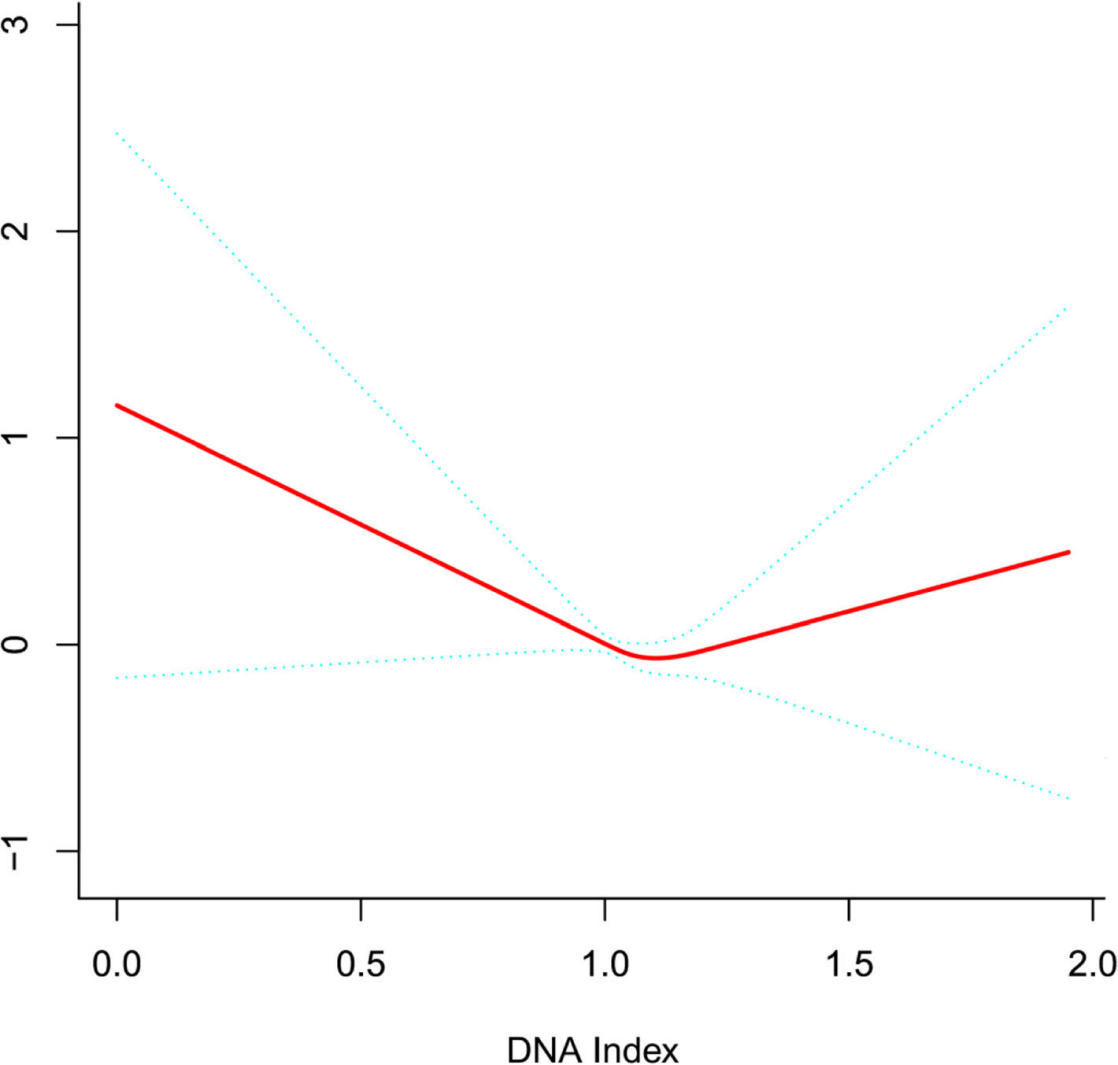
General additive models demonstrate the relationship between DI and the risk of poor outcome. The resulting figures show the predicted log (relative risk) in the y-axis and DI in the x-axis after adjusting for gender, age, race, chemotherapy protocol, WBC, CNS status, fusion gene, prednisone response, BM blasts on Day+ 29, immunophenotype and karyotype

Generalized additive models(Fig. 2) was used to visually assess functional relationships between DI and the risk of adverse clinical outcomes. This analysis was conducted using both logarithmic transformed and untransformed data. Log (relative risk) can be converted to a relative risk by taking antilog. For example, a log (relative risk) of 0 implies the relative risk of 1 (no impact on the probability of having adverse clinical outcomes), whereas a log (relative risk) of 1 implies the relative risk of 2.71 (ie, 2.71-fold increase in the probability of having adverse clinical outcomes). After adjusting for these possible factors related to adverse clinical outcomes, including gender, age, race, chemotherapy protocol, WBC, CNS status, fusion gene, prednisone response, BM blasts day+ 29, immunophenotype and karyotype, the U-shaped relationships between DI and adverse clinical outcomes were confirmed in multivariate analyses. The threshold effect of DI on poor outcome was significant after adjusting for potential confounders. The adjusted regression coefficient (Log RR) was 0.7 (95%*CI* 0.1–3.2, *P* = 0.597) for DI < 1.1 while 8.8 (95%*CI* 1.4–56.0, *P* = 0.021) for DI ≥ 1.2 and 0.0 (95%*CI* 0.0–0.8, *P* = 0.041) for 1.1 ≤ DI < 1.2 (Table 4). Analyses showed that the lowest rates of the adverse outcomes estimated to occur among DI between 1.1 and 1.2. Moreover, a poor outcome significantly increased with increasing DI after the turning point (DI ≥ 1.2)(Fig. 2).

**Table 4:** Threshold effect analysis of DI on adverse clinical outcomes using piece-wise linear regression

DI	Crude		^a^Adjust	
	*LogRR*(95%*CI*)	*P* value	*LogRR*(95%CI)	*P* value
< 1.1	0.6 (0.1, 2.9)	0.559	0.7 (0.1, 3.2)	0.597
≥1.1, < 1.2	0.0 (0.0, 1.7)	0.077	0.0 (0.0, 0.8)	0.041
≥1.2	2.8 (0.5, 15.3)	0.236	8.8 (1.4, 56.0)	0.021

Crude:none adjustment

^a^Adjusted: gender; age; race; chemotherapy protocol; WBC; CNS Status; fusion gene; prednisone response; BM blasts on Day+ 29; immunophenotype; karyotype

### Survival analysis of DI cut-point

**Fig. 3:**
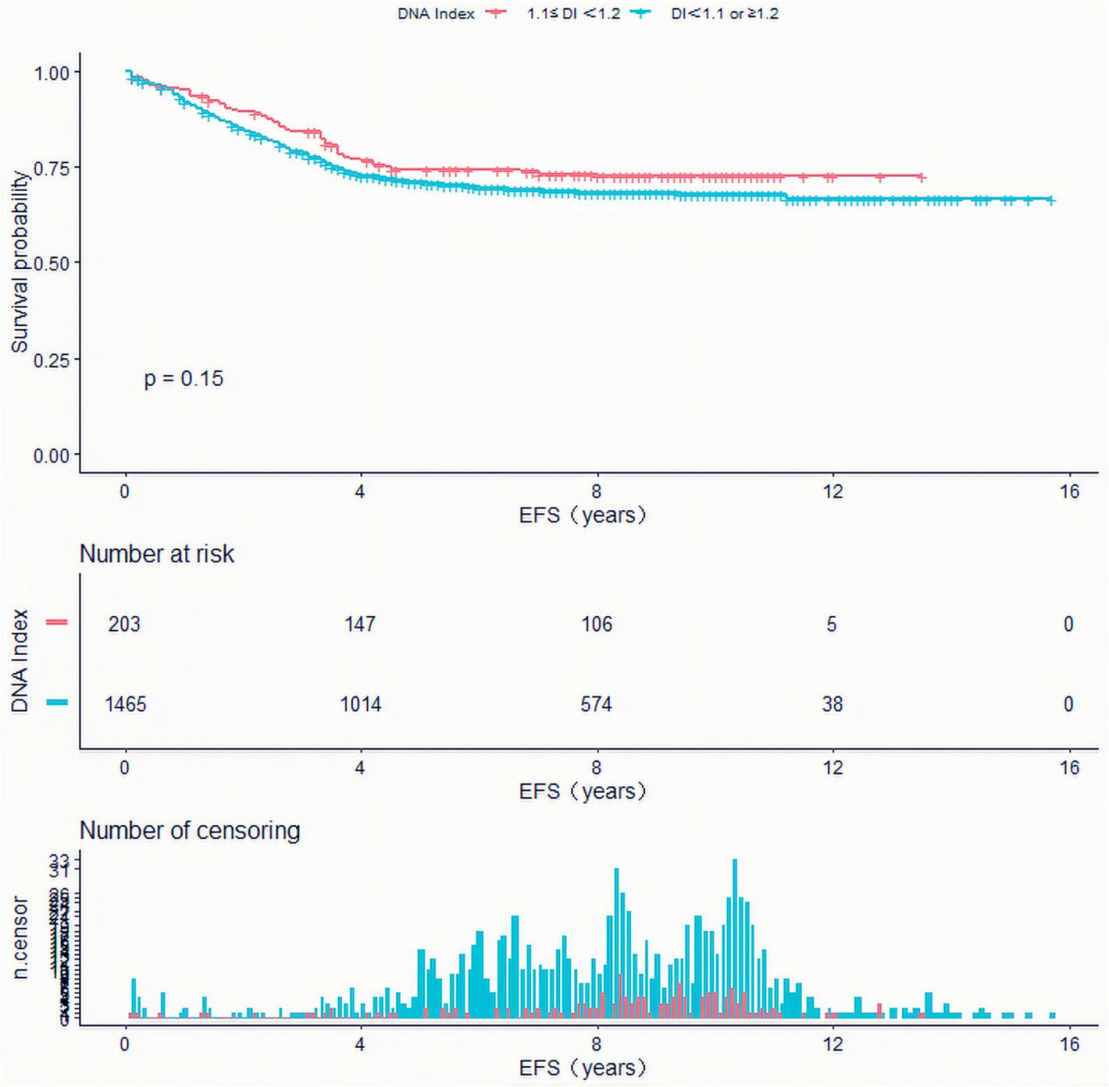
Event-free survival of ALL pediatric patients with DNA index (DI) of 1.1 ≤ DI<1.2, and DI of < 1.1 or ≥ 1.2

**Fig. 4:**
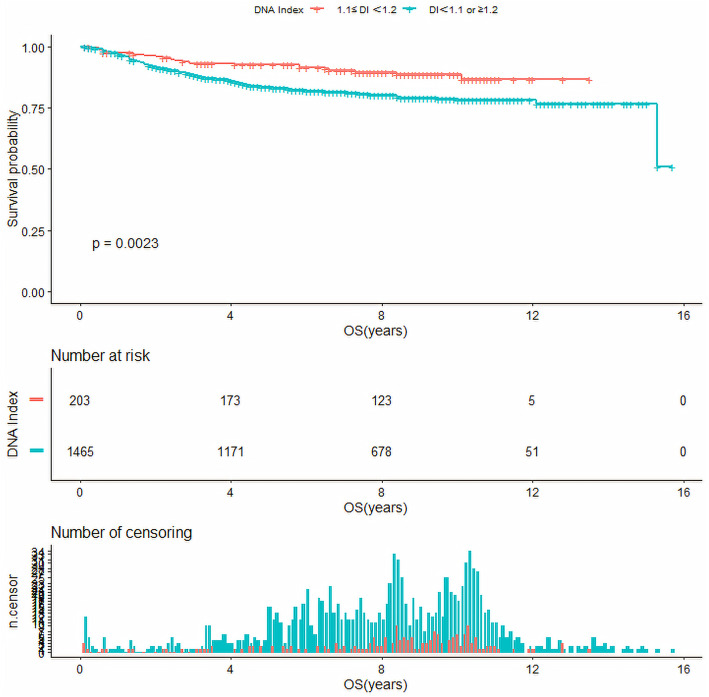
Overall survival of ALL pediatric patients with DNA index (DI) of 1.1 ≤ DI<1.2, and DI of < 1.1 or ≥ 1.2

Base on DI cut-point of 1.1 and 1.2 obtained above, we analyzed the survival as just three groups having different DI ranges (DI < 1.1, 1.1 ≤ DI < 1.2 and DI ≥ 1.2). As a result, the EFS of pediatric ALL with a DI between 1.1–1.2 were higher than those with DI of < 1.1 or ≥ 1.2 (10-year EFS, 72.6 ± 6% versus 67.7 ± 2%, *P* = 0.15), but no significant difference was found. However, the OS of pediatric ALL with a DI between 1.1–1.2 were significantly higher than those with DI of < 1.1 or ≥ 1.2 (10-year OS, 88.9 ± 4% versus 78.3 ± 3%, *P* < 0.05)(Fig. 3, Fig. 4).

## Discussion

The TARGET ALL project team consists of several Children’s Oncology Group(COG) investigators from different institutions. After the inclusion and exclusion criteria, 1668 pediatric ALL were eventually enrolled in our study, which was the largest-scale study to evaluate the value of the DI among childhood ALL treated with recent chemotherapy protocols since 2000s.

First of all, we investigated the value of DI in early treatment response of childhood ALL. In the existing evaluation system, the main factors affecting the early treatment response of childhood ALL include age, initial WBC, specific gene fusions and so on. According our univariate and multivariate analysis, which showed that age ≥ 10 years, BCR/ABL1 fusion gene and poor prednisone response had a significant negative impact on early treatment response, while hyperdiploid and an improved chemotherapy regimen had a significant positive impact, and these results were basically consistent with those reported in previous literature [16–20]. It has been reported [21] that a low DNA index was beneficial for early treatment response. However, no association was found between DI and early treatment response. Based on the above results, we implied those low DI population might both had higher WBC and older age that lead to the correlation was not obvious.

ETV6/RUNX1 positive ALL is considered to occur in prenatal period, which may precede the preleukemic stage [22]. Furthermore, the existence of this fusion transcript changes the differentiation process of hematopoietic progenitor cells and enhances the self-renewal of hematopoietic progenitor cells, especially B-line hematopoietic progenitor cells [23]. Wang [24] et al. reported that the induced remission rate of 77 ETV6/RUNX1 positive B-ALL children was 100%, and the 5-year EFS and OS were 90 ± 3 and 96% ±3%, respectively. Based on the favorable molecular response to treatment and good clinical outcomes, this rearrangement is considered to be of significant therapeutic significance. Results of multivariate analysis from our study showed ETV6/RUNX1 fusion gene significantly associated with better EFS and OS. These findings were consistent with a pediatric study of previous studies [24–26]. It is known to all that BCR/ABL positive is a factor of poor prognosis among childhood ALL. In the current study, we not only further confirmed that the positive expression of this gene leads to poor response to early treatment, but also closely related to the survival rate of pediatric ALL. In addition, BM NR on Day+ 29 indeed decreased the EFS and OS among the children.

Moreover, we showed an independent favorable outcome for the patients on DI in terms of 10-year EFS and OS.We further revealed a threshold effect based on the DI and provide clear evidence of a nonlinear association between DI and adverse clinical outcomes. To the best of our knowledge, this is the first study to describe a U-shaped relationship between DI and adverse clinical outcomes. Interestingly, using these data, we were able to identify DI ranging from 1.1 to 1.2 in which the rates of an adverse clinical outcome of induction failure, relapse, progression or death of any cause were lowest in our population. Next, we explored the survival as just three groups having different DI levels among pediatric patients with ALL. Finally we found that DI between 1.1 to 1.2 conferred favorable prognostic impact on survival in patients with DI of <1.1 or ≥ 1.2. As for the EFS was not significantly different in the 1.1–1.2 group, but the OS was much different and statistically significantly. We supplied that there were many factors that affect EFS, but the only factor that affects OS was whether the patient was death.

Inconsistent with previous studies [6–9], DI of ≥1.16 were not a significantly associated with high EFS and OS in our study but DI of 1.1–1.2 did. In addition, Noh [10] et al. reported that the group with a DI of 1.00–1.90 showed significantly higher OS and EFS than the group with a DI of < 1.00 or > 1.90, whereas the DI of 1.16 was not a significant cut-point in discriminating the risk group among children.We speculated that the reason for the inconsistency with our results may be that the number of cases included in Noh’s studies was too small and was limited to pre-B. Secondly, our pediatric patients were treated with the recent chemotherapy protocol. Furthermore, we used smooth curve fitting after adjusting the confounding factors, which greatly increases the reliability of our results. Taken together, our study revealed that the DI of 1.1–1.2 was a significant cut-point to evaluate the prognostic value among childhood ALL, although pediatric patients were treated with treatment protocols. This information will be useful in the application of evidence that those DI out of 1.1–1.2 should targeted adjustment of chemotherapy program, reduce complications, and pay attention to close follow-up.

The study has several limitations. Most importantly, it was a retrospective study, so bias in this study was inevitably. Secondly, the lack of some data may lead to the incompleteness of the results. In spite of these limitations, this study is a multicenter, large-scale retrospective study to re-evaluate the value of DI, which provide strong evidence for those pediatric ALL treated with recent chemotherapy protocols. In conclusion, the DI between 1.1 and 1.2 can serve as a significant cut-point discriminating the risk group as ever, which remained an independent prognostic factor.

## Data Availability

The data sets used and/or analysed during the current study are available in TARGET dataset (https://ocg.cancer.gov/ programs/target/data-matrix).
